# Root and rhizosphere traits for enhanced water and nutrients uptake efficiency in dynamic environments

**DOI:** 10.3389/fpls.2024.1383373

**Published:** 2024-07-31

**Authors:** Maire Holz, Mohsen Zarebanadkouki, Pascal Benard, Mathias Hoffmann, Maren Dubbert

**Affiliations:** ^1^ Landscape Functioning, Leibniz Centre for Agricultural Landscape Research (ZALF), Müncheberg, Germany; ^2^ Soil Biophysics and Environmental Systems, Technical University of Munich (TUM), Freising, Germany; ^3^ Physics of Soils and Terrestrial Ecosystems, Swiss Federal Institute of Technology (ETH), Zurich, Switzerland

**Keywords:** carbon cost, root trait, rhizosphere, dynamic environment, combined stress, plasticity

## Abstract

Modern agriculture’s goal of improving crop resource acquisition efficiency relies on the intricate relationship between the root system and the soil. Root and rhizosphere traits play a critical role in the efficient use of nutrients and water, especially under dynamic environments. This review emphasizes a holistic perspective, challenging the conventional separation of nutrient and water uptake processes and the necessity for an integrated approach. Anticipating climate change-induced increase in the likelihood of extreme weather events that result in fluctuations in soil moisture and nutrient availability, the study explores the adaptive potential of root and rhizosphere traits to mitigate stress. We emphasize the significance of root and rhizosphere characteristics that enable crops to rapidly respond to varying resource availabilities (i.e. the presence of water and mobile nutrients in the root zone) and their accessibility (i.e. the possibility to transport resources to the root surface). These traits encompass for example root hairs, mucilage and extracellular polymeric substance (EPS) exudation, rhizosheath formation and the expression of nutrient and water transporters. Moreover, we recognize the challenge of balancing carbon investments, especially under stress, where optimized traits must consider carbon-efficient strategies. To advance our understanding, the review calls for well-designed field experiments, recognizing the limitations of controlled environments. Non-destructive methods such as mini rhizotron assessments and *in-situ* stable isotope techniques, in combination with destructive approaches such as root exudation analysis, are proposed for assessing root and rhizosphere traits. The integration of modeling, experimentation, and plant breeding is essential for developing resilient crop genotypes capable of adapting to evolving resource limitation.

## Introduction

Sustainable food production relies on the efficient acquisition and utilization of soil resources by crops. Key to this challenge is the plants root system which controls the access to essential resources like nutrients and water. Traditionally, the prevailing perspective has been to treat nutrient and water uptake as distinct processes. Crucially, however, we argue that both processes are closely linked by the underlying physical mechanisms that control soil transport properties and related processes.

Plant water and nutrient accessibility are significantly impacted by soil water content, particularly the cross-section of water-filled pores and their spatial distribution within the soil matrix. Nutrient transport within soils and to the roots is driven by mass flow (e.g. nitrogen in form of nitrate) and diffusion (e.g. phosphorus) (Marschner, 2011). Mass flow depends on both the movement of water within the soil and the availability of nutrients in the soil solution. Nutrient diffusion is driven by concentration gradients and is controlled by the diffusion properties of the soil, which are highly dependent on soil water content (Barber, 1962). The relative importance of mass flow and diffusion in nutrient transport to roots is nutrient-specific, depending on the mobility and availability of each nutrient within the soil (Tester and Leigh, 2001). However, the effective transport of most nutrients, those transported by diffusion and by mass flow to roots, is significantly hindered as soil dries (Seiffert et al., 1995; Zarebanadkouki et al., 2019). In this review, we therefore emphasize the interconnected nature of water and nutrient uptake processes and advocate for a holistic view that considers the interplay of root and rhizosphere traits that facilitate both nutrient and water acquisition. In this context, availability refers to the presence of water and mobile nutrients, while accessibility pertains to the transport of these resources to the root surface. By adopting an integrated perspective, we can better understand and optimize the root-soil interactions that are critical for sustainable crop production in the face of variable and often limited soil moisture conditions.

Growing concerns suggest climate change will intensify perturbations in soil moisture (Eyring et al., 2021). This, in turn, disrupts the availability and accessibility of water and nutrients for plants (Mitchell et al., 2006; Rahmstorf and Coumou, 2011). Consequently, predicting both the amount and variability of soil water will become more challenging, further complicating the picture of nutrient availability. Primary factors like precipitation and temperature strongly influence soil microbial activity which in turn affects nutrient turnover and availability (Schimel, 2018). We argue that the expected rise in fluctuating soil water and nutrient levels requires the development of genotypes that can adapt to these conditions.

A promising strategy for breeding plants with superior nutrient and water efficiency lies in harnessing root and rhizosphere traits (Wissuwa et al., 2009; Lynch, 2019; Rangappa et al., 2023; Galindo-Castañeda et al., 2024). Root characteristics can be categorized into architectural (e.g. rooting depth, branching), morphological (e.g. root hairs), physiological (e.g. nutrient transporters), and biotic (symbiosis with microorganisms) (Bardgett et al., 2014). These traits significantly impact a plant’s ability to explore the soil, mobilize nutrients, and absorb resources (e.g., Ma et al., 2001; Lynch, 2013; Griffiths and York, 2020). Additionally, rhizosphere traits describe the properties of soil in the vicinity of roots (Wissuwa et al., 2009) modified by root exudation, root-microbial interactions or changes in soil structure. Root and rhizosphere traits influence the accessibility and availability of water and nutrients in soil. While water and nutrient availability is controlled by factors like soil texture, structure, organic matter, pH and microbial activity (Droogers et al., 1997; Fageria and Baligar, 2005), accessibility, determined by soil moisture and transport properties, reflects the ease with which plants can acquire these resources to meet transpiration and nutritional needs (Bray, 1954; Droogers et al., 1997).

As extreme events and environmental patterns become more frequent, a crucial question emerges: which plant traits are most suited to mitigate the adverse effects induced by these changes? This ability, known as plasticity, allows organisms to adjust their physical characteristics (phenotype) based on the environment (Sultan, 2000; Yetgin, 2023). However, the usefulness of plasticity in responding to rapidly changing environments depends on how quickly a trait can be adapted. For example, establishing symbiosis with mycorrhiza is a gradual process with delayed benefits, and initial stages may temporarily hinder plant growth (Plenchette et al., 2005; Jacott et al., 2017). Consequently, this symbiosis might be particularly advantageous in situations characterized by static stress conditions. Additionally, many traits are known to enhance resource acquisition of singular resources (Lynch and Brown, 2001; Lynch, 2013). As such “topsoil foraging” has been suggested as a root architectural ideotype for phosphorus limiting conditions, including for example lateral root branching, shallow rooting angles or high crown root numbers (Lynch and Brown, 2001; Zhu et al., 2005; Sun et al., 2018). In contrast, the “steep, cheap and deep” ideotype has been suggested for water and nitrogen limiting conditions including traits like low crown root numbers, steep rooting angles and low lateral branching (Klein et al., 2000; Lynch, 2013, 2018). The inherent risk of optimization conflicts, for example if deep rooting during drought conditions results in diminished nutrient uptake from shallow soil, have been addressed recently for root traits (Van Der Bom et al., 2020; Rangarajan et al., 2022). However, the role of rhizosphere traits in the context of combined water and nutrient stress has received little attention in the past.

To tackle rapidly fluctuating climatic conditions that lead to dynamic soil moisture and nutrient limitations, we need to focus on root and rhizosphere processes that offer 1) rapid (temporal scale of seconds to days) responses beneficial for 2) both, water and nutrient supply. These could be traits that do not change quickly but enable the plant to react fast (deep rooting and subsequent water uptake change from deeper layers or hydraulic lift) or traits that respond fast to changing resource availabilities (exudation, root hairs). For example, a critical feature to enable a crop to rapidly respond to drying soil is to adjust root water uptake proportionality and to redistribute water horizontally (hydraulic lift) (Horton and Hart, 1998). Hydraulic lift serves the dual purpose of facilitating both the movement of deep water and the absorption of nutrients by roots from superficial soil layers, where, in many cases, nutrients, such as phosphorus, are more abundant. However, rooting depth, root axial hydraulic conductivity and xylem diameter, the underlying traits enabling hydraulic lift or changes in root water uptake proportionality, do not display rapid plasticity (Bechmann et al., 2014; Vadez, 2014).

There is a significant need for novel analytical frameworks in order to assess the impact of root and rhizosphere traits holistically. While progress has been made in understanding the impact of different root and rhizosphere traits on water and nutrient uptake, significant gaps remain, especially with regard to field conditions. This is primarily related to the complexity of the rhizosphere, the manifold feedbacks shaping it, and the upscaling of small scale processes.

This review explores root and rhizosphere traits allowing crops to rapidly respond to environmental perturbation and adjust to challenging variations in water and nutrient conditions simultaneously. Moreover, we propose a novel observational scheme to quantify the impact of root/rhizosphere traits on water and nutrient co-limitation under field conditions.

## Root and rhizosphere traits in dynamic resource limited environments

**Figure 1:**
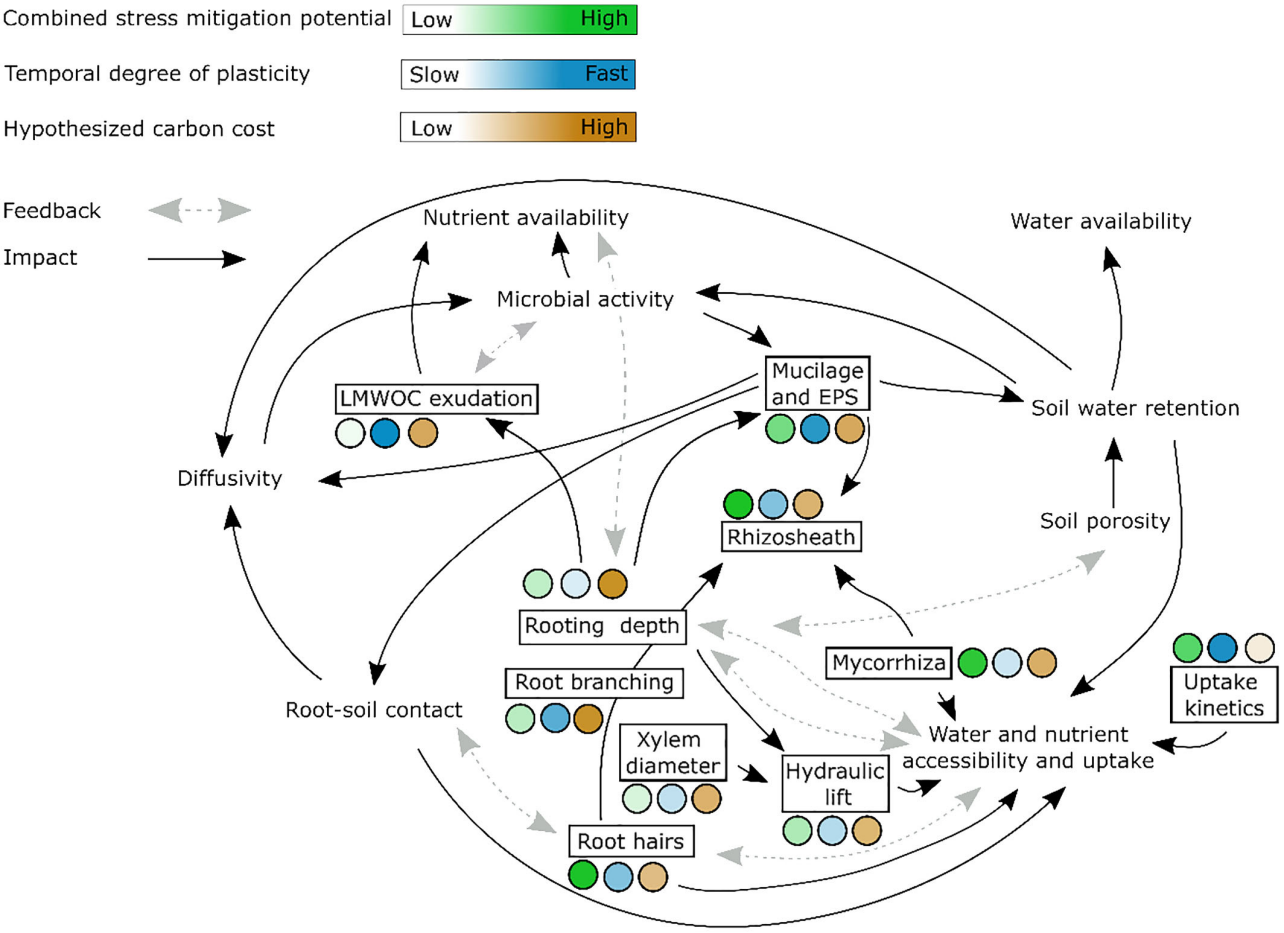
Root and rhizosphere traits (displayed in black boxes) that are suggested to enhance water and nutrient uptake efficiency in dynamic environments and that are covered by this review. We here distinguish water/nutrient availability and accessibility. Availability refers to the amount and forms of water and nutrients that are present for plant uptake while accessibility is determined by soil moisture and transport properties and reflects the ease with which plants can acquire these resources. The color gradients indicate the ability of traits to help acquiring water and nutrients under combined stress (green), the temporal degree of plasticity, i.e. how fast traits can be adjusted to changing environments (blue) and the carbon costs of the traits (brown). Note that the information on carbon cost is hypothetical, as for many traits this has not been quantified but can rather be assumed or estimated. LMWOS: low molecular weight organic substances.

Traits that exhibit rapid responsiveness to changing environmental conditions and that enhance water and nutrient supply simultaneously are predominantly observed within morphological root traits and rhizosphere features. The most promising root and rhizosphere traits to simultaneously enhance water and nutrient uptake efficiency in dynamic environments are summarized in **Figure 1**. One example are root hairs that are well known to enhance nutrient acquisition and exhibit highly plastic and potentially quick response to resource limitation (Nestler and Wissuwa, 2016; Klamer et al., 2019). Root hairs are efficient due to their ability to increase effective root surface area and forage for nutrients (Bienert et al., 2021; Rongsawat et al., 2021). Additionally, root hairs are identified as hotspots for root exudation, promoting microbial and enzymatic activities (Holz et al., 2020, 2017). In contrast, the contribution of root hairs to water uptake is debated, with less consistent results on their effectiveness in sustaining plant transpiration during drought (Carminati et al., 2017; Cai et al., 2021; Marin et al., 2021; Duddek et al., 2022). Therefore, while being a promising root trait with rapid and substantial plastic responses, the effectiveness of root hairs in alleviating combined water and nutrient stress requires further research.

Disentangling the impact of changes in water and nutrient fluxes at the rhizosphere scale becomes exceptionally challenging. As the soil dries, both the hydraulic conductivity and the diffusion of solutes, which are crucial for transport, diminish (Javaux and Carminati, 2021). Consequently, under dry conditions, rhizosphere properties may limit plant transpiration and nutrient uptake (Lang and Gardner, 1970; Carminati et al., 2017). Critical rhizosphere traits like root exudation and rhizosphere soil structure, however, play a vital role in enhancing the availability and accessibility of water and nutrients to plants.

Root exudation of low molecular weight carbon (LMWOC) compounds is a rhizosphere trait that is often associated with plant nutrient acquisition (Dakora and Phillips, 2002). Particularly organic anions are well known for their P solubilization and plastic response upon P limitation (Zhang et al., 1997; Neumann and Römheld, 1999). When it comes to the combined water and nutrient stress, mucilage, a gel-like substance released from root tips, is a promising trait. Mucilage possesses three intrinsic properties crucial for its functional impact on water dynamics in the soil: the ability to retain water, to reduce water’s surface tension, and to increase water viscosity (e.g. Read and Gregory, 1997; Benard et al., 2019). Additionally, a positive effect on P solubilization has been suggested (Grimal et al., 2001; Liebersbach et al., 2004). Due to mucilage’s characteristics, it can help maintaining a wet rhizosphere during soil drying and improve the contact between roots, soil and soil water, thereby ensuring sustained water and nutrient transport (Carminati et al., 2010; Holz et al., 2019; Zarebanadkouki et al., 2019). Additionally, enhanced liquid retention promotes microbial activity in the rhizosphere by providing more stable conditions for solute diffusion and protection from desiccation (Or et al., 2007; Nazari et al., 2020). The extent to which plants can exhibit plastic responses and adapt mucilage production remains unclear. However, considering that mucilage secretion is an active process (Weston et al., 2012), it is reasonable to speculate that plants might engage in such plastic responses. Similarly, extracellular polymeric substances (EPS) released by microorganisms can induce comparable modifications of soil transport properties (Benard et al., 2019; Zarebanadkouki et al., 2019). This is of fundamental importance as microbial activity is controlled by the diffusion of enzymes and nutrients (Schimel, 2018), particularly in the rhizosphere. Improved diffusion benefits rhizosphere microorganisms in two ways: a sustained supply of root metabolites, and enhanced nutrient availability and accessibility through improved enzymatic activity and solute transport. However, understanding the feedback between soil moisture, nutrient conditions, soil properties, microorganisms, and roots in shaping rhizosphere hydraulics requires further research.

The complex interaction between different substances such as mucilage and EPS but also root hairs, lead to soil structural changes, resulting in the so called rhizosheath formation (Mo et al., 2023; Pang et al., 2017). The rhizosheath is defined as the weight of soil adhering to the root surface upon excavation (McCully, 1999). Though no correlation between root hair length and rhizosheath weight has been found in previous studies, the presence of root hairs is required for effective rhizosheath formation (Brown et al., 2017). Additionally, mucilage and microbial EPS are suggested to control rhizosheath formation (McCully, 1999; Pang et al., 2017; Mo et al., 2023). Rhizosheath creates a protective microenvironment around roots, enhancing water and nutrient absorption by improving soil structure, fostering microbial activity, and facilitating the release and utilization of root exudates (Cheraghi et al., 2023). Rhizosheath formation starts with root penetration, altering soil porosity and initiating its structural dynamics (Helliwell et al., 2017). It is thereafter controlled by soil moisture fluctuations and EPS/mucilage acting as glueing agents enhancing soil structural stability (Martin, 1971; Czarnes et al., 2000). Note that rhizosheath, as a quantity is ill-defined because the water content upon root excavation is rarely controlled nor reported. Yet, since it is an easily obtainable parameter, rhizosheath should be considered as a potential breeding target under combined water and nutrient stress (Cheraghi et al., 2023).

Physiological root traits such as aquaporin and nitrate transporter expression (i.e. uptake kinetics) become crucial when soil nutrient availability is unpredictable, and adaptations through changes in root structure are challenging for plants (Jackson and Caldwell, 1996; Schneider and Lynch, 2020). Yet, when it comes to physiological traits marked by high plasticity, substantial knowledge gaps persist, given their comparatively limited exploration compared to better-studied aspects like root architecture (Griffiths and York, 2020). When combined with rhizosphere traits that enhance nutrient and water availability, physiological responses such as increased transporter activity could have a substantial positive impact on the plant. However, the interaction between traits such as mucilage or EPS exudation and physiological traits such as transporter expression needs further investigation.

## Root-shoot interaction and links with carbon metabolism

When identifying plastic traits enhancing both nutrient and water uptake, it’s crucial to recognize that 1) the root system and its plasticity is not acting isolated from the aboveground (Mao et al., 2018) and 2) that the cost efficiency of carbon investments might significantly change under drought stress or nutrient limitation (Wang et al., 2021).

Consequently, overarching strategies of plants to optimize water use describe an arsenal of below- and aboveground, physiological and morphological adaptations working in conjunction to mitigate drought (Cowan, 1982; Anderegg et al., 2018). A useful classification is the distinction between isohydric (“conservative water use strategy”) with early stomatal closure upon soil drying at the expense of reduced carbon gain, and anisohydric (less conservative water use strategy maintaining high stomatal conductance and carbon gain at the risk of hydraulic failure under drought) (Jones and Sutherland, 1991; McDowell et al., 2008). Isohydric plant species often rely on deep roots and continuous water access. In contrast anisohydric plant species are often shallow rooted (McDowell et al. 2008) but have developed a broad range of structural and functional adaptations (e.g. preventing excessive transpiration by thick cuticle or adjusting leaf angle), allowing them to sustainably function even under severe drought stress (Tardieu and Simmoneau, 1998). Resource utilization strategies are not limited to adaptions of the root system or the rhizosphere but also encompass adaptations to the aboveground plant development: resource acquisitive species reduce the leaf area while increasing their photosynthetic rate or display enhanced nutrient absorption capabilities (Wright et al., 2004; Freschet et al., 2010; Reich, 2014; Díaz et al., 2016).

Hence, breeding for effective drought and nutrient adaptation faces the challenge of linking below- and aboveground plant responses. Furthermore, investing in root and rhizosphere traits may compromise carbon allocation to aboveground biomass in agricultural plants, potentially affecting yields (Jansson et al., 2021). Under stress, especially when photosynthesis is limited, balancing water, nutrient, and carbon metabolism becomes challenging. Developing (plastic) traits in response to drought and nutrient limitations can be costly in terms of carbon investment, particularly when carbon availability is limited. Therefore, optimizing traits should encompass both carbon-efficiency and prioritization of carbon assimilation or the photosynthesis/respiration balance (Poorter and Nagel, 2000; Amthor et al., 2019). Up to now, information on the carbon costs of most root and rhizosphere traits remains limited. While we hypothesize about these costs based on physiological principles (as reflected in **Figure 1**), quantifying the exact energetic investment for various traits is a developing field. This highlights the importance of further research in this area.

## Way forward

This review stresses the need to view nutrient and water uptake as interconnected rather than independent processes. Climate change, with increased variability in soil moisture and nutrient conditions, requires genotypes capable of swift adjustments to changing environmental conditions. Examining phenotypic plasticity in root and rhizosphere traits and rapid responses of crops under combined resource limitations provides insights into their adaptive potential. In particular, root morphological and physiological root traits, along with rhizosphere features, allow for high responsiveness to combined water and nutrient limitations at the root-soil interface.

To study these promising root and rhizosphere traits, future research needs well-designed experimental setups, preferably in authentic field conditions, as controlled conditions may differ from real-world settings, impacting transferability (Nestler et al., 2017). To account for dynamic environmental conditions in response to the accelerating climate change, we propose conducting drought-recovery experiments rather than solely relying on static drought stress scenarios (Zheng et al., 2023).

**Figure 2:**
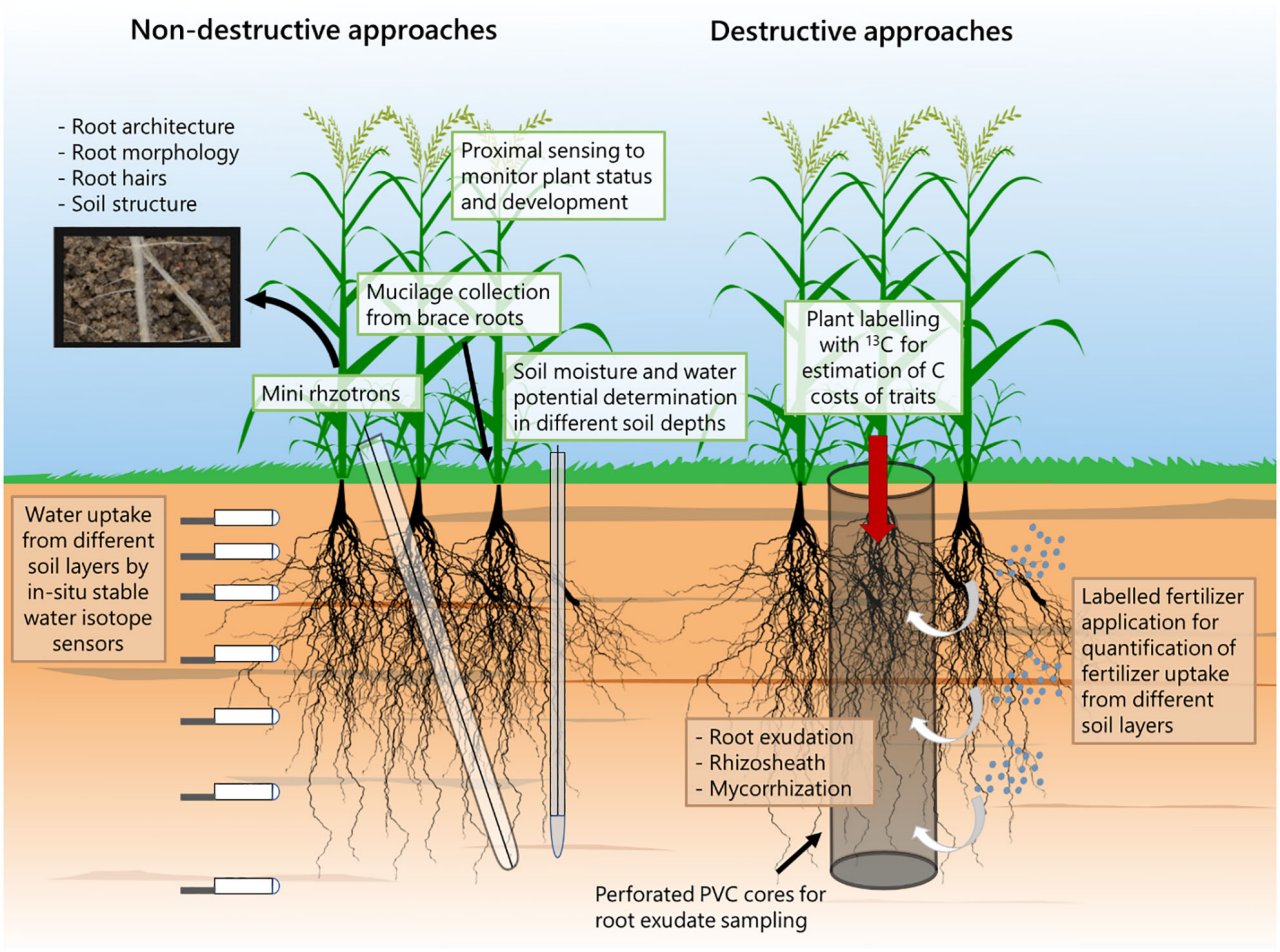
Suggested approaches that can be used in combination in order to study root and rhizosphere traits for combined water and nutrient limitation.

To capture a range of valuable root and rhizosphere traits, we suggest using diverse measurement approaches (**Figure 2**). Non-destructive methods can be used, such as measuring water potentials along the soil-plant-atmosphere gradient (Turner et al., 1984; Pagay, 2022) and estimating water uptake from varying soil depths by combining *in-situ* stable water isotope sensors and statistical modelling (Deseano Diaz et al., 2023; Dubbert et al., 2023).

Mini rhizotron assessments enable the quantification of root morphology, including root hairs and, to some extent, root architecture across various soil depths over time (Vamerali et al., 2012; Vincent et al., 2017). Another viable method is mucilage collection from brace roots, especially in maize plants (Ahmed et al., 2015).

Finally, proximal sensing techniques, like spectral reflectance and infrared thermometry, non-invasively monitor plant status and development (Karthikeyan et al., 2020; Kimaro et al., 2023), allowing real-time assessments of biomass production, canopy structure, and photosynthetic activity (Gamon et al., 2015). Continuous monitoring of spectral reflectance during the growth period provides insights into physiological status, enabling early detection of nutrient limitations (Weiss et al., 2020; Fiorentini et al., 2021; Sanaeifar et al., 2023), possibly guiding breeding programs towards genotypes with improved resource use strategies. Proximal sensing therefore not only contributes to biomass quantification but also enhances our capacity to proactively address nutrient-related challenges and optimize agricultural practices for improved crop performance and resource use efficiency (Rajath et al., 2021).

Destructive approaches, particularly those for studying the rhizosphere, often involve sampling the entire plant. To collect root exudates in field conditions, perforated columns can be inserted, employing a soil-hydroponic-hybrid exudation sampling approach (Santangeli et al., 2024) (**Figure 2**). We propose combining this approach with a) the assessment of C costs of root traits based on either ^13^C labeling (Zhou et al., 2022) or C3-C4 vegetation change (Kumar et al., 2016) and b) the addition of isotopically labeled fertilizers and irrigation at different soil depths within the perforated columns to determine nutrient uptake from various soil layers (Kristensen and Thorup-Kristensen, 2004; Chen et al., 2021).

Integrating insights gained from these diverse measurement approaches with efforts in plant breeding (Galindo-Castañeda et al., 2024; Wissuwa et al., 2009), can help accelerate the development of crop varieties specifically designed to thrive under future climate scenarios.

This holistic approach ensures a comprehensive understanding of root and rhizosphere traits under diverse conditions, guiding future agricultural strategies for sustainable resource management.

## Author contributions

MH: Conceptualization, Visualization, Writing – original draft. MZ: Conceptualization, Writing – original draft. PB: Conceptualization, Visualization, Writing – original draft. MH: Conceptualization, Visualization, Writing – original draft. MD: Writing – original draft.
